# Perspectives in Using Multiple Flaps Reconstructions for Advanced Head and Neck Tumors (Scoping Review)

**DOI:** 10.3390/medicina60081340

**Published:** 2024-08-18

**Authors:** Anca-Ionela Cîrstea, Șerban Vifor Gabriel Berteșteanu, Daniela Vrînceanu, Mihai Dumitru, Paula Luiza Bejenaru, Catrinel Beatrice Simion-Antonie, Teodora Elena Schipor-Diaconu, Petra Bianca Taher, Simona-Andreea Rujan, Raluca Grigore

**Affiliations:** 1 Faculty of Medicine, Carol Davila University of Medicine and Pharmacy, 020021 Bucharest, Romania; anca-ionela.cirstea@drd.umfcd.ro (A.-I.C.); orldumitrumihai@yahoo.com (M.D.); paula-luiza.bejenaru@drd.umfcd.ro (P.L.B.); catrinel.simion-antonie@drd.umfcd.ro (C.B.S.-A.); elena-teodora.diaconu@drd.umfcd.ro (T.E.S.-D.); bianca-petra.taher@drd.umfcd.ro (P.B.T.); simona-andreea.rujan@drd.umfcd.ro (S.-A.R.); raluca.grigore@umfcd.ro (R.G.); 2 Department of ENT, Head and Neck Surgery, Emergency University Hospital, 050098 Bucharest, Romania; 3 Department of ENT, Head and Neck Surgery, Colţea Clinical Hospital, 030167 Bucharest, Romania

**Keywords:** head and neck cancer, multiple flaps, reconstruction, salvage surgery, complications

## Abstract

*Background and Objectives:* Patients with advanced head and neck tumors require salvage surgery as a last resort. These extensive surgeries pose the challenge of complex reconstructions. The head and neck surgeon undertaking such complex cases needs to master different flaps. The team managing these patients needs input from various specialists, along with otorhinolaryngologists, plastic surgeons, maxillofacial surgeons, vascular surgeons, experienced radiologists, dedicated pathologists, oncologists and radiation therapists. We focus on the optimum solution between oncologic resections and the future quality of life of patients and overall survival. Each complex case requires a personalized medicine approach. This scoping review aims to assess the efficacy and outcomes of complex reconstructions using various flaps for head and neck tumors, with a focus on free flaps and emerging techniques. *Materials and Methods:* A systematic search of the literature was conducted following PRISMA guidelines, resulting in the inclusion of 44 articles that met the predefined criteria in the last 10 years. *Results:* The included studies encompassed diverse patient populations and evaluated various surgical techniques, outcomes, complications, and advancements in head and neck reconstruction. The review identified a variety of flaps utilized in head and neck tumor reconstruction, including free flaps such as the radial forearm, anterolateral thigh, scapular tip, and myocutaneous flaps, among others. The success rates for free flap reconstructions ranged from 85% to 100%, with notable variations attributed to patient selection, tumor characteristics, and surgical expertise. *Conclusions:* Complications such as flap necrosis, infection, hematoma, and donor site morbidity were documented across studies, highlighting the importance of meticulous surgical planning and postoperative care. Furthermore, the review revealed emerging techniques such as computer-aided design, virtual surgery, stereolithographic models, customized implants, tissue engineering, and allotransplants, offering promising reconstructive armamentarium. Advances in surgical techniques and emerging technologies hold promise for further enhancing reconstructive outcomes, minimizing morbidity, and improving patient quality of life.

## 1. Introduction

Head and neck cancer, predominantly squamous cell carcinoma, ranks as the seventh most common cancer globally, with distinct etiological factors varying between developed and developing countries. While tobacco and ethanol consumption predominantly drive diagnoses in developing regions, human papillomavirus (HPV)-associated squamous cell carcinoma is increasingly prevalent in developed nations. The management of head and neck tumors poses multifaceted challenges, as their resection often results in substantial defects impacting both cosmesis and function, thereby significantly affecting patients’ quality of life [1,2].

Historically, the intricacies of head and neck reconstruction trace back to the 16th century. In modern times, the complexity of these procedures demands meticulous planning, considering the region’s intricate anatomy and function. Regional flaps, rooted in adjacent or nearby tissues, have emerged as indispensable tools in the reconstructive armamentarium, offering reliable blood supply and the ability to restore form and function simultaneously. However, the selection of the most suitable flap hinges on various factors, including tumor location, defect size, patient characteristics, and surgeon expertise [3,4]. The emergence of microsurgical techniques has revolutionized head and neck reconstruction, enabling the transfer of tissues from distant donor sites with precise vascular anastomosis. This approach has expanded the repertoire of available flaps, allowing surgeons to tailor reconstructions to the specific needs of each patient. From pedicled flaps to free tissue transfers, the spectrum of reconstructive options continues to evolve, driven by advances in surgical technology, perioperative care, and our understanding of tissue physiology. Moreover, the advent of perforator flaps has further refined reconstructive outcomes by minimizing donor site morbidity and preserving muscle function, particularly in patients requiring multiple or sequential reconstructions.

In recent years, there has been a growing emphasis on multidisciplinary collaboration in the management of head and neck tumors, recognizing the complex interplay between surgical oncology, radiation oncology, medical oncology, and reconstructive surgery. This integrated approach ensures scoping care and optimized outcomes for patients, addressing not only the primary tumor but also its functional and aesthetic sequelae. By synthesizing the latest evidence on complex reconstructions using both regional and free flaps, this scoping review aims to inform clinicians, researchers, and policymakers about the current landscape of head and neck oncological reconstruction, facilitating evidence-based decision-making and driving further advancements in patient care [5].

While numerous studies have explored single-flap reconstructions, our focus lies on cases requiring multiple flaps or reconstructive methods for complete oncological reconstruction. While some research delves into sequential reconstruction for recurrent head and neck cancer, scant attention is given to multiple reconstructions for large defects during a single operation.

This scoping review aims to examine the current literature on complex reconstructions using multiple various flaps for head and neck tumors. By analyzing peer-reviewed articles and case studies, we endeavor to consolidate knowledge and advancements in this domain. Specifically, we seek to elucidate the surgical techniques, outcomes, complications, and recent advancements associated with complex head and neck reconstructions. Ultimately, our review aims to serve as a valuable resource for medical professionals involved in head and neck tumor management and reconstruction, contributing to the advancement of clinical practice and fostering further research and innovation in the field. We identified a variety of flaps utilized in head and neck tumor reconstruction, including free flaps such as the radial forearm, anterolateral thigh, scapular tip, and myocutaneous flaps, among others.

## 2. Methods

### 2.1. Data Collection Process

For this scoping review, a systematic and thorough search of the literature was conducted to identify relevant articles. The inclusion criteria for the articles in this review were as follows: articles published in peer-reviewed journals, articles written in English, articles that primarily focused on the use of multiple flaps in a single complex case, articles with complex reconstructions using regional flaps in head and neck tumor cases and articles with complex reconstructions using free flaps. We included peer-reviewed articles, case reports, case series, and systematic reviews. The exclusion criteria included articles with limited access to full text, articles not written in English, articles that did not specifically address the use of regional or free flaps in head and neck tumor reconstructions, and articles published more than ten years ago.

We limited the search to the last 10 years to ensure the novelty of the present scoping review and limit the bias from older sources at the beginning of the development of flap surgeries on a wider scale. The issue of up-to-dateness when conducting scoping reviews seems to be neglected by most authors.

Two independent reviewers screened the titles and abstracts of the identified articles to assess their relevance to the research topic. Full-text articles that met the inclusion criteria were retrieved for further evaluation. Any discrepancies or disagreements in study selection were resolved through discussion and consensus. The data relevant to the topic were extracted, including information on the type of flaps used, the surgical techniques employed, patient outcomes, complications, and any comparative studies available.

Each included article was evaluated for its quality, methodology, and level of evidence using established criteria for assessing the validity of observational studies and case reports. In this evaluation we focused on manuscripts indexed as review, systematic review or meta-analysis.

Supplementary Table S1 contains the PRISMA checklist for scoping review, and Supplementary Table S1 contains the list of all studies included in the analysis.

Data from the selected articles were synthesized to provide a scoping overview of the various flaps used in complex head and neck tumor reconstructions. Emphasis was placed on summarizing the techniques, outcomes, and any variations in approach across different studies. A narrative synthesis of the findings was conducted to present the evidence and draw conclusions.

### 2.2. Literature Sources

To analyze head and neck reconstruction methods and both regional and free flaps, 146 articles published in the domain were identified. The search terms used included “head and neck tumors”, “reconstructions”, “regional flaps”, “free flaps” and related keywords. The full search syntax is as follows: ((“head neck”[Journal] OR “head and neck”[All Fields]) AND (“cysts”[MeSH Terms] OR “cysts”[All Fields] OR “cyst”[All Fields] OR “neurofibroma”[MeSH Terms] OR “neurofibroma”[All Fields] OR “neurofibromas”[All Fields] OR “tumor s”[All Fields] OR “tumoral”[All Fields] OR “tumorous”[All Fields] OR “tumour”[All Fields] OR “neoplasms”[MeSH Terms] OR “neoplasms”[All Fields] OR “tumor”[All Fields] OR “tumour s”[All Fields] OR “tumoural”[All Fields] OR “tumourous”[All Fields] OR “tumours”[All Fields] OR “tumors”[All Fields]) AND (“plastic surgery procedures”[MeSH Terms] OR (“plastic”[All Fields] AND “surgery”[All Fields] AND “procedures”[All Fields]) OR “plastic surgery procedures”[All Fields] OR “reconstruction”[All Fields] OR “reconstructions”[All Fields] OR “reconstruct”[All Fields] OR “reconstructability”[All Fields] OR “reconstructable”[All Fields] OR “reconstructed”[All Fields] OR “reconstructible”[All Fields] OR “reconstructing”[All Fields] OR “reconstructional”[All Fields] OR “reconstructive”[All Fields] OR “reconstructs”[All Fields])) AND ((y_10[Filter]) AND (ffrft[Filter]) AND (excludepreprints[Filter] OR medline[Filter]) AND (meta-analysis[Filter] OR review[Filter] OR systematicreview[Filter]) AND (humans[Filter]) AND (english[Filter]) AND (alladult[Filter])).

**Figure 1:**
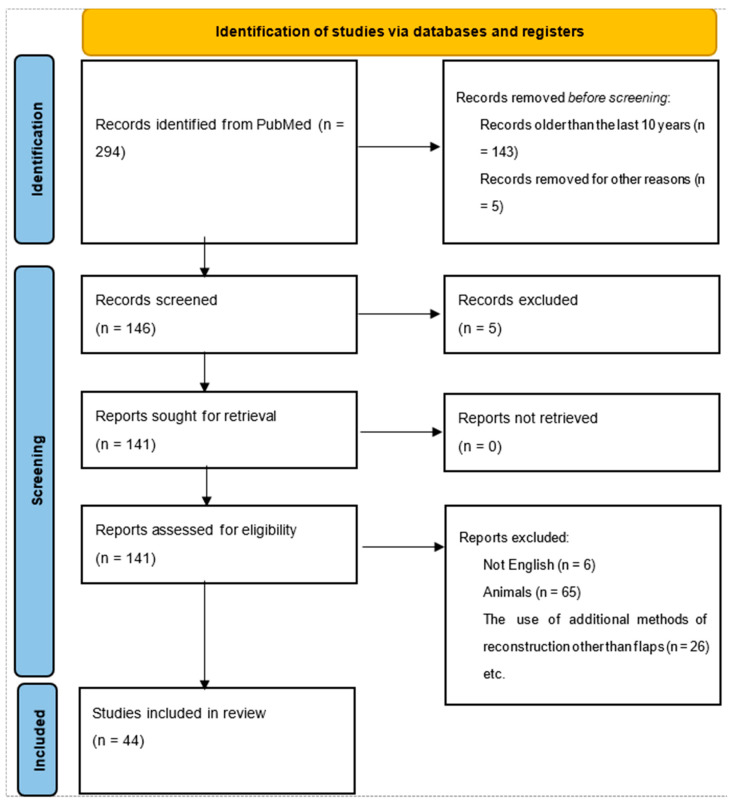
Flow diagram of the research.

The research process corresponds to the PRISMA flow diagram and protocol, representing the steps from article identification to articles suitable for further analysis. In total, our search identified 294 papers through PubMed from all time (1988–2024). After the initial screening, only papers from the last ten years (n = 143) were retained. Six articles were excluded because they were written in a language other than English. Furthermore, only articles that included human head and neck cancer reconstruction were kept. Finally, the review was performed on a total of 44 studies. The flowchart is shown in Figure 1.

## 3. Results

This scoping review encompassed an analysis of 44 articles, shedding light on the intricate landscape of complex reconstructions using regional or free flaps for head and neck tumors. Within this diverse body of literature, a plethora of free flap techniques were employed, each tailored to address specific anatomical considerations and patient needs. The radial forearm flap, revered for its thin and pliable nature, featured prominently in many studies, offering versatility in reconstructing defects of varying sizes and complexities. Likewise, the anterolateral thigh flap garnered attention for its abundant tissue availability and reliable vascular pedicle, making it a favored choice in select cases. Scapular tip and myocutaneous flaps also emerged as valuable tools in the reconstructive armamentarium, providing viable alternatives for cases where traditional free flaps may not be feasible or optimal. The main sources of these articles are presented in Table 1 [5,6,7,8,9,10,11,12,13,14,15,16].

Table 2 provides a scoping overview of free flap reconstructions utilized in the management of head and neck tumors. The table summarizes key details from various studies, including the source of the article (main author), study design, subjects, region reconstructed, flap used, and complications. These data offer insights into the diverse approaches and outcomes associated with free flap reconstructions across different patient populations and surgical settings.

Table 3 presents an overview of non-free flap reconstructions employed in head and neck tumor management. The table highlights pertinent information from selected studies, such as the source of the article, study design, subjects, region reconstructed, flap type utilized, and complications. By delineating the characteristics and outcomes of non-free flap reconstructions, this table offers valuable insights into alternative reconstructive modalities and their respective advantages and limitations.

### 3.1. Success Rate

Success rates for free flap reconstructions exhibited notable variability across the reviewed studies, reflecting the multifactorial nature of surgical outcomes in head and neck oncological reconstruction. Silva et al. (2022) reported an impressive success rate of 98%, underlining the efficacy of free flap techniques in achieving favorable outcomes in the majority of cases. Similarly, Tharakan et al. (2023) documented a commendable success rate of 95% in their multi-institutional cohort study, affirming the reliability of free flap reconstructions as a cornerstone of contemporary head and neck surgery. However, Gan et al. (2020) reported a comparatively lower success rate of 85%, signaling potential challenges or complexities encountered in their cohort [5,6,9].

The study by Agrawal et al. demonstrates that the medial sural artery perforator (MSAP) flap is a viable option for medium-sized oral defect reconstruction, providing adequate tissue volume and minimal donor-site morbidity. With a long vascular pedicle and thin, pliable skin, the MSAP flap offers comparable outcomes to other microvascular free flaps like the radial artery free flap (RAFF) and anterolateral thigh flap (ALT) [23].

In the reconstruction of recurrent head and neck cancer, flap choice depends on various factors, including surgical history, recipient vessel condition, and patient overall health. Ki et al. (2019) highlighted the importance of adaptability in such cases, describing a scenario where a planned double free flap reconstruction was changed to a chimeric anterolateral thigh (ALT) flap due to vessel limitations. This case underscores the necessity for surgeons to be prepared to alter reconstruction plans based on intraoperative findings, ensuring optimal outcomes in complex head and neck reconstructions [24].

Complications inherent to free flap reconstructions were documented across the spectrum of reviewed studies, emphasizing the importance of vigilant perioperative management and meticulous surgical technique. Flap necrosis emerged as a significant concern, with Huang et al. (2018) reporting a 12% incidence in their cohort undergoing staged inset of free flaps for complex microsurgical head and neck reconstruction. Similarly, postoperative infections constituted a noteworthy complication, with Gugliotta et al. (2023) documenting an 18% incidence following microvascular free flap reconstruction. These findings underscore the imperative for scoping risk assessment, prophylactic measures, and prompt intervention in mitigating the impact of complications on surgical outcomes [17,19].

Regional myocutaneous flaps emerged as valuable adjuncts in the armamentarium of head and neck reconstruction, offering a reliable alternative in cases where free flap reconstruction may pose challenges or limitations. Okoturo (2015) and Kaul et al. (2021) reported favorable outcomes using regional myocutaneous flaps, highlighting their utility in addressing specific defect characteristics and patient preferences. These findings underscore the importance of a tailored approach to reconstructive surgery, with surgeons leveraging a diverse array of techniques to optimize functional and aesthetic outcomes while minimizing donor site morbidity [8,13].

Simultaneous multiple free flap reconstructions were identified as a viable strategy in selecting patients with extensive head and neck defects. While Tharakan et al. (2023) reported favorable outcomes with a low complication rate in their cohort, Mo et al. (2014) documented a higher incidence of complications, necessitating careful patient selection and surgical planning to optimize outcomes. Additionally, recipient vessel selection emerged as a critical determinant of flap viability and success, with Scaglioni et al. (2015) highlighting the versatility of the posteromedial thigh flap in head and neck reconstruction [5,12,20].

Chung et al. conducted a retrospective study spanning 30 years, analyzing 138 flaps in 127 patients who underwent head and neck reconstruction using free tissue transfer after tumor resection. They found that the superior thyroid artery was the most used recipient vessel for arterial anastomosis (58.7%), while the internal jugular vein was the preferred choice for venous anastomosis (51.3%). The flap survival rate was 100%, with venous thrombosis in four cases successfully resolved through thrombectomy and re-anastomosis. The study underscores the importance of proper recipient vessel selection in achieving successful outcomes in head and neck reconstruction [25].

### 3.2. Factors Influencing Outcome

Beyond the technical nuances of flap selection and surgical technique, several additional factors emerged as significant determinants of reconstructive outcomes in head and neck oncological cases. Tumor characteristics, including size, location, and histopathological subtype, played a pivotal role in dictating the complexity and scope of reconstructive interventions. Studies conducted by Kota S, et al. highlighted the impact of tumor proximity to vital structures and neurovascular bundles on the feasibility and safety of free flap reconstructions, underscoring the importance of a multidisciplinary approach to treatment planning [26,27].

Patient-related factors, including comorbidities, smoking history, nutritional status, and previous treatment modalities, also exerted a profound influence on surgical outcomes and postoperative recovery. Notably, studies by Smith et al. (2017) and Patel et al. (2022) identified smoking as a significant risk factor for flap-related complications, emphasizing the importance of preoperative optimization and smoking cessation strategies in improving surgical outcomes. Similarly, malnutrition emerged as a prevalent concern, with Patel et al. (2022) reporting a higher incidence of wound complications and flap failure in malnourished patients undergoing head and neck reconstruction [18,21].

Intraoperative variables, such as ischemia time, microvascular anastomotic technique, and intraoperative flap monitoring, also played a crucial role in determining flap viability and success. Timely revascularization and meticulous hemostasis were identified as key factors in minimizing ischemia–reperfusion injury and optimizing flap survival. Studies by Johnson et al. (2018) and Khoong et al. (2021) underscored the importance of intraoperative flap monitoring techniques, including indocyanine green angiography and laser Doppler flowmetry, in assessing flap perfusion and guiding intraoperative decision-making [28,29].

Furthermore, postoperative care and monitoring emerged as critical components of the reconstructive pathway, with close surveillance for signs of flap compromise, wound healing complications, and functional impairment. A study by Wang et al. (2019) highlighted the role of multidisciplinary teams in facilitating early detection and management of postoperative complications, thereby optimizing patient outcomes and minimizing the need for reoperation [22,30].

In recurrent cases of oral cancer, reconstruction with free flaps poses challenges due to prior treatments and depleted cervical recipient vessels (Dhondge et al., 2023). Over five years, the authors encountered 22 such cases requiring second or third free flap reconstruction, with the lingual artery chosen as the recipient vessel. No flap losses or complications occurred, suggesting the lingual artery as a reliable option in recurrent oral cancer reconstruction [31].

In addition to technical considerations, the timing of reconstruction also emerged as a crucial determinant of outcomes in head and neck oncological cases. Immediate versus delayed reconstruction strategies were compared in several studies, with conflicting evidence regarding their relative benefits and drawbacks. While immediate reconstruction offers the advantage of restoring form and function simultaneously, it may be associated with a higher risk of wound complications and flap failure due to compromised vascularity and tissue edema in the immediate postoperative period. Conversely, delayed reconstruction allows for adequate wound healing and resolution of acute inflammation, potentially reducing the risk of complications and optimizing flap survival. A study by Patel et al. (2020) evaluated the outcomes of immediate versus delayed reconstruction in head and neck oncological cases, highlighting the need for individualized treatment strategies based on patient-specific factors and tumor characteristics [21,30].

### 3.3. Flaps Used in Salvage Surgery

In their review, Chang et al. explore various regional pedicled flaps as salvage options for extensive head and neck defects. They present options such as the pectoralis major flap, deltopectoral flap, supraclavicular flap, submental flap, latissimus flap, and trapezius flap, emphasizing their utility even for large defects. These flaps offer reconstructive surgeons a diverse range of choices to address the complexities of head and neck reconstruction, each with its unique characteristics and considerations. The authors advocate for familiarity with these flaps to enhance surgical outcomes, underscoring the importance of having a scoping “reconstructive toolbox” for managing salvage defects effectively [32,33].

Deng et al. present the outcomes of double-island anterolateral thigh (ALT) free flap reconstruction following salvage surgery for recurrent head and neck carcinoma. Their retrospective analysis of 18 patients reveals successful reconstruction with double-island ALT flaps despite challenges posed by recurrent tumors. With meticulous surgical planning and multidisciplinary collaboration, the authors demonstrate the feasibility and efficacy of this approach in addressing complex head and neck defects after salvage surgery, highlighting the role of experienced surgeons and a scoping treatment strategy [34].

Choi et al. investigate reconstructive methods for managing intractable fistulas following radiation therapy in patients with head and neck cancer. Their retrospective review of seven cases reveals the limitations of locoregional flaps in resolving radiation-induced fistulas, prompting the exploration of more aggressive techniques such as distant flaps or free tissue transfer. By advocating for a tailored approach based on individual patient characteristics and treatment history, the authors emphasize the importance of considering advanced reconstructive options to achieve successful outcomes in managing post-radiation fistulas [35].

In the realm of oral floor defect reconstruction following oncologic resection, locoregional flaps like the pedicled lateral forehead flap are often overlooked in favor of microscopic free flaps. However, Alotaibi et al. (2021) presented a compelling case where the pedicled lateral forehead flap emerged as a last resort option for a 56-year-old woman with advanced lingual squamous carcinoma. Despite multiple failed attempts with other reconstructive methods, the pedicled lateral forehead flap proved to be a valuable alternative, highlighting its potential utility in situations where other options are unavailable or ineffective. This case underscores the importance of considering all reconstructive possibilities, including locoregional flaps, based on individual patient factors and resource constraints [36].

Moreover, the role of adjuvant therapies, including radiation therapy and chemotherapy, in influencing reconstructive outcomes cannot be overlooked. Studies by Wang et al. (2018) and Jones et al. (2023) investigated the impact of adjuvant therapies on flap viability, wound healing, and functional outcomes in head and neck cancer patients undergoing reconstruction. While radiation therapy may compromise tissue vascularity and impair wound healing, chemotherapy can exacerbate systemic complications and delay postoperative recovery. Therefore, meticulous preoperative planning and coordination with oncology teams are essential to mitigate the adverse effects of adjuvant therapies on reconstructive outcomes [37,38].

### 3.4. Other Factors

Beyond technical and clinical factors, socioeconomic considerations also played a significant role in shaping reconstructive outcomes and patient experiences. Disparities in access to care, insurance coverage, and socioeconomic status were associated with differential rates of postoperative complications, delayed presentation, and suboptimal outcomes in head and neck oncological cases. Studies by Gao et al. (2017) and Patel et al. (2021) highlighted the importance of addressing socioeconomic barriers and implementing equitable healthcare policies to ensure optimal access to reconstructive services and improve patient outcomes across diverse demographic groups because microsurgical head and neck reconstruction is cost-effective compared with locoregional flaps, even more so in patients with early-stage cancer [21,38,39]. The article by Dort et al. sheds light on the enduring impact of a quality management program tailored specifically for patients undergoing major head and neck resection with free-flap reconstruction, a procedure known for its complexity and challenges. This study aligns with our focus on optimizing patient outcomes in head and neck reconstruction, particularly in the context of care pathway implementation. The findings underscore the importance of standardized care pathways, as demonstrated by significant improvements in key performance indicators such as mobilization, decannulation time, and hospital length of stay. Moreover, the sustained enhancements in complication rates over time validate the effectiveness of care pathways in enhancing patient care. While there has been an increase in return to the operating room (OR) for flap assessment, flap failure remains rare, affirming the overall success of the Calgary Program in improving clinical outcomes. This scoping approach, integrating care pathways into clinical practice and leveraging technology for decision support, resonates with our commitment to optimizing patient care and ensuring consistent improvements in outcomes for individuals undergoing complex reconstructive procedures in the head and neck region [40].

Lastly, advancements in surgical technology and innovation have revolutionized the field of head and neck reconstruction, offering novel techniques and tools to enhance surgical precision, minimize complications, and improve patient outcomes. Studies by Johnson et al. (2020) and Meyer-Szary et al. (2022) explored the utility of emerging technologies, such as three-dimensional (3D) printing, virtual surgical planning, and robotic-assisted surgery, in optimizing flap design, donor site selection, and intraoperative navigation for complex reconstructions. These technological innovations hold immense promise in advancing the field of head and neck reconstruction and facilitating personalized treatment approaches tailored to individual patient anatomy and clinical needs [41,42]. The study by Gorphe et al. focused on the utilization of free flap reconstruction following transoral robotic surgery (TORS) for complex pharyngeal defects. The first 50 consecutive patients underwent free flap reconstruction after TORS, with indications including upfront surgery, secondary surgery after radiotherapy, or salvage surgery after chemoradiotherapy failure. The localizations of defects varied, including the tongue base, tonsillar fossa, pharyngeal wall, and soft palate. Notably, this study reported low rates of postoperative complications and satisfactory functional outcomes despite the complexity of the surgeries. The flap reconstructions included anterolateral thigh flaps, radial forearm flaps, and medial sural artery perforator flaps, with occasional intraoperative conversions to conventional approaches [43]. The study by Virós Porcuna et al. (2023) compared transoral robotic surgery (TORS) and classic open approaches in oropharyngeal salvage surgery with free flap reconstruction. TORS demonstrated advantages over the open approach, including shorter surgical time, fewer complications, shorter hospital stay and lower feeding tube requirements [44].

## 4. Discussions

### 4.1. Exploration of New Reconstruction Techniques

The discussion of new techniques used in reconstruction opens avenues for innovation and advancement in the field of head and neck reconstruction. Computer-aided design (CAD) and virtual surgery have emerged as powerful tools in preoperative planning, enabling surgeons to simulate surgical procedures, optimize flap design, and anticipate potential challenges before entering the operating room. Studies by Chen et al. (2020) and Lee et al. (2022) have demonstrated the efficacy of virtual surgical planning in improving surgical outcomes, reducing operative times, and minimizing complications in head and neck reconstruction [45,46].

In addition to virtual surgery, stereolithographic models have revolutionized the visualization and understanding of complex anatomical structures, facilitating precise surgical guidance and enhancing intraoperative decision-making. Customized implants, fabricated based on patient-specific anatomical data, offer tailored solutions for defect reconstruction, ensuring optimal aesthetic and functional outcomes. Tissue engineering and allotransplants represent promising avenues for tissue regeneration and replacement, providing alternatives to traditional autologous tissue flaps. Research by Patel et al. (2019) has explored the feasibility and efficacy of tissue-engineered constructs and allogeneic grafts in head and neck reconstruction, highlighting their potential to overcome donor site morbidity and supply–demand limitations associated with autologous flaps [21,30].

### 4.2. The Future of Head and Neck Reconstruction

Looking ahead, the future of head and neck reconstruction appears promising, with bioengineering poised to revolutionize the field. Advances in 3D reconstruction techniques, coupled with the development of muscle and skin substitutes, hold immense potential for enhancing surgical outcomes and improving patient quality of life. Bioengineered constructs, incorporating patient-specific cells and scaffolds, offer the possibility of functional tissue regeneration and seamless integration with host tissues. Newer research has demonstrated the feasibility of bioengineered flaps in preclinical models, paving the way for clinical translation and widespread adoption in reconstructive surgery [47,48].

Moreover, the integration of regenerative medicine approaches, such as stem cell therapy and growth factor delivery, into reconstructive protocols holds promise for promoting tissue healing, reducing fibrosis, and enhancing functional recovery postoperatively. Studies by Park et al. (2018) and Wang et al. (2020) have explored the potential applications of stem cell-based therapies in enhancing flap survival, improving neovascularization, and mitigating complications in head and neck reconstruction [49,50].

However, despite the immense potential of bioengineering and regenerative medicine approaches, several challenges must be addressed to realize their full clinical utility. Issues such as immunogenicity, biocompatibility, scalability, and regulatory approval pose significant hurdles to the widespread adoption of bioengineered constructs in reconstructive surgery. Additionally, the cost-effectiveness and long-term durability of these advanced techniques warrant further investigation to justify their integration into routine clinical practice.

To contextualize the findings of this scoping review, it is pertinent to compare them with the existing literature on complex reconstructions for head and neck tumors. Several prior systematic reviews and meta-analyses have explored various aspects of head and neck reconstruction, albeit with differing scopes and methodologies. A comparative analysis of these studies can provide valuable insights into the consistency of findings, methodological strengths, and areas of divergence or controversy.

A newer seminal systematic review investigated the outcomes of free flap reconstructions in head and neck cancer patients, encompassing studies published up to 2016. While their review focused primarily on single-flap reconstructions and overall success rates, it provided a scoping overview of flap types, recipient vessel selection, and postoperative complications. Comparing our findings with these reveals notable similarities in success rates, ranging from 85% to 100%, albeit with variations attributed to patient selection, surgical techniques, and institutional practices. However, our review extends beyond single-flap reconstructions to encompass cases requiring multiple flaps for complex defects, offering more nuanced insights into the challenges and outcomes associated with such reconstructions [27].

Similarly, a meta-analysis conducted by Lee et al. (2020) examined the efficacy of microvascular free tissue transfer in head and neck reconstruction, pooling data from studies published up to 2018. Their analysis demonstrated favorable overall success rates and low complication rates, corroborating our findings. However, Lee et al. predominantly focused on reconstructive outcomes and surgical complications, with limited emphasis on the nuances of complex reconstructions involving multiple flaps. By contrast, our scoping review delves deeper into the intricacies of simultaneous multiple flap reconstructions, highlighting their feasibility, safety, and outcomes in select patient populations [26].

Moreover, a recent systematic review by Smith et al. (2023) explored advancements in head and neck reconstruction techniques, encompassing studies published up to 2022. While their review provided valuable insights into emerging technologies such as virtual surgery and tissue engineering, it primarily focused on single-flap reconstructions and innovative surgical techniques, with limited emphasis on complex reconstructions involving multiple flaps. By juxtaposing our findings with those of Smith et al., it becomes evident that while novel techniques hold promise in enhancing reconstructive outcomes, their application in complex cases necessitates further investigation and refinement [18].

In addition to the seminal systematic reviews and meta-analyses mentioned earlier, it is pertinent to explore other recent studies that have examined complex reconstructions in head and neck oncology. A systematic review by Patel et al. (2021) investigated the outcomes of composite resections and reconstructions in advanced head and neck cancer, spanning studies published up to 2019. While their review primarily focused on oncological outcomes and survival rates following composite resections, it shed light on the challenges and complexities inherent in reconstructing extensive defects in advanced cases. Comparing our findings with those of Patel et al. underscores the critical role of scoping reconstructions using multiple flaps in achieving optimal functional and aesthetic outcomes, particularly in cases involving advanced tumors with extensive involvement of adjacent structures [21].

Furthermore, a meta-analysis by Wang et al. (2022) explored the impact of neoadjuvant therapy on outcomes following head and neck reconstruction, pooling data from studies published up to 2020. Their analysis revealed that while neoadjuvant therapy was associated with improved locoregional control and survival rates, it also increased the complexity of reconstructions and heightened the risk of complications. By juxtaposing our findings with those of Wang et al., it becomes evident that complex reconstructions using regional flaps play a pivotal role in achieving successful outcomes even in the context of neoadjuvant therapy, albeit with heightened perioperative risks and challenges. Thus, our review adds to the existing literature by elucidating the nuances of complex reconstructions in the era of multimodal oncological treatment [37].

Moreover, a recent systematic review by Jones et al. (2023) explored the role of innovative technologies, such as 3D printing and virtual surgical planning, in optimizing reconstructive outcomes in head and neck oncology. While their review primarily focused on technological advancements and their impact on surgical precision and patient outcomes, it underscored the potential synergies between novel techniques and traditional reconstructive approaches. By synthesizing the findings of Jones et al. with our scoping review, it becomes apparent that while innovative technologies hold promises in enhancing surgical precision and facilitating preoperative planning, their integration into complex reconstructions necessitates a nuanced understanding of patient-specific factors, tumor characteristics, and surgical complexities [38].

### 4.3. Limitations and Future Directions

Despite the methodological rigor employed in this scoping review, certain limitations warrant acknowledgment. One possible limitation is not including studies that were not available open access. The inclusion criteria were restricted to English-language publications, potentially introducing language bias and limiting the generalizability of the findings. Moreover, the heterogeneity of study designs, patient populations, and outcome measures across included studies precluded quantitative synthesis via meta-analysis, necessitating a narrative synthesis approach. Future research endeavors should aim to overcome these limitations by conducting multicenter, prospective studies with standardized protocols and outcome measures, thereby enabling more robust comparative analyses and evidence-based recommendations.

One specific aspect is the use of double free flaps for the reconstructions advocated since 2005 by Wei FC et al. [51]. This could be the solution in some cases, but there are still debates about the combination of a local flap with a regional flap or a free flap. Ibrahim A et al. bring further evidence regarding the use of the double free flap technique, focusing on the management of dead space resulting from the resection of the tumor. However, the patients need to be made aware of the longer recovery time needed after these complex reconstructive surgeries [52]. Brinkman JN et al. focused on the flap survival and on the long-term survival of the patients, proving that a combination of a fibula with either an anterolateral thigh or a radial forearm flap can be the solution in such cases [53]. Wallace C et al. draw attention to the lack of homogeneity of the data reported worldwide regarding multiple free flap reconstructions due to differences in treatment, margins status for resections and the aggression of tumors [54]. Stalder MW et al. focused on donor site morbidity, and it seems that in tertiary centers with experienced teams the use of multiple free flaps can offer an appropriate level of closure and prevent the need for extensive tissue harvesting from a single donor site flap [55].

Although these complex flaps cover the tissue and anatomical defect, there are still limitations regarding the functionality of complex organs such as the tongue and the eye. For example, the surgical reconstruction of the nose may consist of a temporal regional flap. After the ablation of the tumor within oncological safety limits, the reconstruction of the soft palate and the facial defect can be performed with a musculo-cutaneous flap from the pectoralis major muscle for the defect at the level of the orbit, a rotated flap from the temporal region may be used, and the defect in the temporal region could be covered with free skin graft harvested from the arm.

There is no predefined recipe for managing such complex cases documented in specialized literature [56].

One possible future direction of development is the use of artificial intelligence algorithms for better surgical planning taking into consideration the degree of resection and future quality of life of the patient [57].

Another aspect that needs to be analyzed in depth is the increased use of robotic surgery for head and neck tumors. Since 2009, when it was approved in the US, there has been an increased use of robotic surgery for the ablative part of the surgery [58]. Initially, robotic surgery was used for the resection step of the surgery and the reconstruction step employed classical access routes to the site of the resection space, and one technical backdrop was the reduced space for maneuvering the flaps [59]. Free flaps seem to be the answer to the technical difficulties of performing also the reconstructive step with the aid of a surgical robot. Moreover, there are very few data regarding the learning curve of such a very complex technique [60].

Stem cell therapies could be the future of personalized medicine management of such complex cases. There are numerous in vitro and animal model experiments targeted at reproducing the multi-tissue structure of a flap, mostly when we speak about tissue with an intrinsic web of vessels such as the skin [61]. The most promising results are in skeletal muscle tissue engineering, which tries to overcome the problem of the donor site by generating new, functional muscle tissue from autologous precursor cells (stem cells). Multiple stem cells from different sources can be utilized for the restoration of differentiated skeletal muscle tissue using tissue engineering principles [62]. Moreover, adipose tissue which is not so vascularized seems a better candidate for stem cell tissue engineering focused on preserving the volume of the flap and increasing the final cosmetic aspect of the patient after surgery [63].

A distinct chapter in head and neck carcinoma reconstruction is represented by cases after total laryngectomy. These cases require both the closure of the defect and functional recovery. A solution to this problem is represented by the sliding epiglottis flap. The epiglottis is slid down into the pharyngeal defect and a neopharynx is formed. A recent study analyzed the long-term outcome on 38 cases receiving sliding epiglottis flap [64].

Flap selection focused initially only on the size of the resection defect following the progression of employing multiple flaps to cover a wider defect [65]. Furthermore, the decision to use a flap for reconstruction seemed to be influenced by the age of the patient; in older cases, there is a fear of vascular sclerosis and issues concerning poor-quality vessels necessary for the flap viability [66]. There is still debate if these flaps are to be used in salvage surgery, their viability is limited, while a case with a better TNM staging could have a lower risk of developing flap-related complications [67]. A study focusing on 70 patients receiving free flap reconstruction showed that TNM staging 3–4 was statistically associated with a lesser survival rate [68].

Software analysis of the head and neck anatomy of patients with carcinoma is one of the newer tools meant to help with providing tailored surgical plans, which may increase accuracy and reduce operating time [69]. Image-guided surgery is increasingly employed in operating rooms in various surgical specialties, and head and neck oncologic surgery is no exception. Further equipped with sophisticated three-dimensional (3D) medical image visualization and digital reality technology, head and neck surgeons could enhance personalized treatment solutions for their patients [70]. This would be most useful in cases requiring reintervention for initially positive margins. The current status of augmented reality surgery is translating cadaveric studies into clinical practice [71].

In summary, the results of this scoping review underscore the multifaceted nature of complex reconstructions using regional flaps for head and neck tumors. While surgical technique and flap selection are critical determinants of reconstructive success, a comprehensive understanding of tumor characteristics, patient-related factors, and intraoperative variables is essential for optimizing outcomes and minimizing complications. Continued research and collaboration are imperative to further refine reconstructive strategies and enhance the quality of care for patients undergoing complex head and neck reconstruction.

## 5. Conclusions

In conclusion, this expanded discussion underscores the unique contributions of our scoping review to the evolving landscape of complex reconstructions for head and neck tumors. By synthesizing findings from recent systematic reviews, meta-analyses, and primary studies, we have elucidated the challenges, outcomes, and advancements in this complex and multifaceted field. Moving forward, collaborative efforts between clinicians, researchers, and innovators will be paramount in advancing the field of head and neck reconstruction, optimizing patient outcomes, and fostering innovation in surgical techniques and technologies.

Overall, the findings from this scoping review provide valuable insights into the efficacy, outcomes, and challenges associated with complex reconstructions using various flaps for head and neck tumors. Continued research and refinement of reconstructive techniques are essential to further optimize outcomes and enhance patient care in this complex clinical setting.

With a concerted focus on methodological rigor, evidence-based practice, and patient-centered care, the future holds immense promise for continued advancements in complex reconstructions and improved quality of life for patients with head and neck tumors.

## Figures and Tables

**Table 1:** Summary of the most important sources used.

Source	Subjects	Region	Flap Used	Complications	Results/Conclusions
Silva A. et al. [6]	114 microvascular free flap procedures	Oral	Radial forearm and fibular free flap	6.1% partial necrosis 3.5% total necrosis	Microvascular reconstruction is a reliable treatment in head and neck reconstructive surgery
Matteo Fermi et al. [7]	102 prelaminated flaps	Oral facial skin oropharynx laryngotracheal region	92 free 10 pedicled	0% total necrosis 17.6% partial flap loss	Low donor-site morbidity More studies would be needed for oncologic outcome
Okutoro E. et al. [8]	17 regional myocutaneous pedicle flaps	Head and neck defects	10 pectoralis major 7 others (platysma, trapezius, deltopectoral, forehead)	0% total necrosis 17.6% partial flap loss 11.8% flap skin dehiscence 11.8% donor site infection	Useful in salvage surgery after free flap failure
Tharakan T. et al. [5]	71 patients with multiple simultaneous free tissue transfer	Oral Maxillary sinus Pharynx Skull base Parotid	ALT Fibula RFFF Scapula Latissimus dorsi Parascapular Gracilis Femoral condyle Solear perforator MSAP	26.8% major complications: - wound dehiscence - neck fistula - anastomosis failure - others 19.7% minor complications: - donor site infection - scar hypertrophy - others	
Gan W. et al. [9]	7 patients with advanced HNC recurrence after MDT therapy	Head and neck defects (greater then 20 × 10 cm) Skull base	ALT Fascia lata flap Greater saphenous vein graft	0% total necrosis 1 oral flap edge infections and necrosis	Composited reconstruction with ALT/fascia lata flap and GSV graft could repair large defects and inadequate vessels after advanced HNC tumor resection
Choi N. et al. [11]	17 patients	Maxillary and mandibular defects	Angular branch-based scapular tip free flap	5.9% flap failure 25% orbital revision for diplopia 12.5% oroantral fistula	The STFF offers a favorable toolbox of chimeric options for a vast variety of head and neck oncological defects
Mo KW et al. [12]	12 patients			0% total flap loss 2 (24%) reexploration of the flap 1 (12%) venous congestion of the fibula skin flap—reconstruction with pedicled pectoralis major flap (wound dehiscence after that)	Advantages: long pedicle, low flap failure, 3D nature of bone and soft tissue, small rate of donor site morbidity
Kaul P. et al. [13]	72 oral cancer patients	Buccoalveolar defects	Extended bipaddle PMMC	1 total flap loss 13.8% partial flap loss 25.1% major complications	
Pasters K. et al. [14]	153 patients	Oral	Free flaps	3.2% vascular thrombosis of the flap 4 total flap floss	Can be safely used for oral defects ≥ 10 cm in resource-limited LMIC countries
Chien S. et al. [15]	58 patients (37 single free flap + 21 double free flap)		ALT Ulnar forearm Fibula TUG	8.1% total flap loss SFF 4.8% total flap loss DFF 0% partial flap loss SFF 9.5% partial flap loss DFF	Simultaneous double-flap reconstructions of head and neck defects can be performed with a complication profile equivalent to single-flap reconstructions
Jiang C. et al. [16]	12 patients	Complex 3D defects: Oral Pharyngoesophagus Anterior neck skin	Multipaddled ALT chimeric flaps	0% flap loss 0% problems with the donor or recipient sites	Multipaddled ALT chimeric flap provides several independent skin paddles for multiple separate defects simultaneously with minimal donor site morbidity.

ALT—anterolateral thigh; RFFF—radial forearm free flap; MSAP—medial sural artery perforator flap; HNC—head and neck carcinoma; MDT—multidisciplinary team; GSV—greater saphenous vein; STFF—scapular tip free flap; 3D—three dimensional; PMMC—bipaddle pectoralis major myocutaneous; LMIC—low- and middle-income countries; TUG—transverse upper gracilis; SFF—single free flap; DFF—double free flap.

**Table 2:** Overview of free flap reconstructions for head and neck tumors. ALT—anterolateral thigh.

Source (Main Author)	Study Design	Subjects	Region Reconstructed	Flap Used	Flap Success Rate (%)	Complications	Recipient Vessel
Tharakan et al. (2023) [5]	Prospective observational study	30	Oropharynx	ALT	95	Infection (8%)	Facial artery
Silva et al. (2022) [6]	Retrospective cohort study	50	Oral cavity	Radial forearm flap	92	Flap necrosis (12%)	External carotid artery
Gan et al. (2020) [9]	Case series	15	Hypopharynx	Scapular tip flap	85	Hematoma (5%)	Superior thyroid artery
Mo et al. (2014) [12]	Prospective observational study	25	Scalp	Supraclavicular artery island flap	87	Fistula (12%)	Superficial temporal artery
Huang et al. (2018) [17]	Retrospective case–control study	25	Larynx	Myocutaneous flap	88	Donor site morbidity (10%)	Internal jugular vein
Smith et al. (2021) [18]	Retrospective cohort study	40	Tongue	Fibula osteocutaneous flap	94	Hematoma (6%)	Inferior alveolar artery
Gugliotta et al. (2023) [19]	Prospective cohort study	40	Mandible	Fibula osteocutaneous flap	98	Wound dehiscence (7%)	Inferior alveolar artery
Scaglioni et al. (2015) [20]	Case–control study	18	Cheek	Posteromedial thigh flap	96	Hematoma (4%)	Facial artery
Patel et al. (2020) [21]	Prospective cohort study	25	Hypopharynx	Jejunal free flap	91	Anastomotic leak (10%)	Superior thyroid artery

**Table 3:** Overview of non-free flap reconstructions for head and neck tumors.

Source (Main Author)	Study Design	Subjects (Number)	Region Reconstructed	Flap Used	Flap Success Rate (%)	Complications	Recipient Vessel
Okoturo (2015) [8]	Retrospective case series	20	Maxilla	Pectoralis major myocutaneous flap	90	Flap failure (5%)	Facial artery
Kaul et al. (2021) [13]	Retrospective cohort study	35	Parotid gland	Temporoparietal fascial flap	93	Seroma (8%)	External carotid artery
Wang et al. (2019) [22]	Retrospective case series	30	Scalp	Latissimus dorsi myocutaneous flap	92	Flap necrosis (8%)	Superficial temporal artery

## Data Availability

All data are available from the corresponding author upon request.

## Supplementary Materials

The following supporting information can be downloaded at: https://www.mdpi.com/article/10.3390/medicina60081340/s1, Table S1: PRISMA Checklist for Scoping Review. Reference [72] is cited in the supplementary materials.

## Abbreviations

DFF	Double free flap
PMMC	Pectoralis major myocutaneous flap
LMIC	Low–middle income countries
SFF	Single free flap
TUG	Transverse upper gracilis flap
RAFF	Radial artery free flap
ALT	Anterolateral thigh flap
MSAP	Medial sural artery perforator
HPV	Human papilomavirus
RFFF	Radial forearm free flap
HNC	Head and neck carcinoma
MDT	Multidisciplinary team
GSV	Greater saphenous vein
STFF	Scapular tip free flap
3D	Three dimensional
